# Influenza Virus Infection in Nonhuman Primates

**DOI:** 10.3201/eid1810.120214

**Published:** 2012-10

**Authors:** Erik A. Karlsson, Gregory A. Engel, M.M. Feeroz, Sorn San, Aida Rompis, Benjamin P. Y.-H. Lee, Eric Shaw, Gunwha Oh, Michael A. Schillaci, Richard Grant, John Heidrich, Stacey Schultz-Cherry, Lisa Jones-Engel

**Affiliations:** St. Jude Children’s Research Hospital, Memphis, Tennessee, USA (E.A. Karlsson, S. Schultz-Cherry);; University of Washington, Seattle, Washington, USA (G.A. Engel, G. Oh, L. Jones-Engel);; Swedish Cherry Hill Family Medicine, Seattle (G.A. Engel);; Jahangirnagar University, Savar, Bangladesh (M.M. Feeroz);; National Veterinary Research Institute, Phnom Penh, Cambodia (S. San);; University of Udayana, Bali, Indonesia (A. Rompis);; Nature Parks, Singapore (B.P.Y.-H. Lee);; Gibraltar Ornithological and Natural History Society, Gibraltar (E. Shaw);; University of Toronto Scarborough, Ontario, Canada (M.A. Schillaci);; and Shin Nippon Biomedical Laboratory, Phnom Penh (R. Grant, J. Heidrich)

**Keywords:** influenza A virus, avian influenza virus, prevalence, nonhuman primates, Macaca, macaque, influenza, viruses, zoonoses

## Abstract

To determine whether nonhuman primates are infected with influenza viruses in nature, we conducted serologic and swab studies among macaques from several parts of the world. Our detection of influenza virus and antibodies to influenza virus raises questions about the role of nonhuman primates in the ecology of influenza.

Worldwide, infections with influenza A viruses are associated with substantial illness and death among mammals and birds. Public health and research have placed major focus on understanding the pathogenicity of different influenza virus strains and characterizing new influenza vaccines. Nonhuman primates (NHPs), including macaques, have become popular experimental models for studying the pathogenesis and immunology of seasonal and emerging influenza viruses. NHPs readily seroconvert after experimental inoculation with seasonal influenza virus and have been used to test candidate vaccines for strains of human and avian origin. Like humans, macaques infected with influenza virus exhibit fever, malaise, nasal discharge, and nonproductive cough; virus replication can be detected in the nasal passages and respiratory tract (*1*,*2*). However, whether NHPs are infected with influenza viruses in nature remains unknown.

Over the past decade, we have focused on the role of pet and performing monkeys in disease transmission throughout Asia. Commonly trapped in the wild, these monkeys might be sold at wet markets, the putative source of several zoonotic outbreaks (*3*), where they might be caged next to any number of animal species (Figure 1, panel A) (*4*). Pet and performing monkeys are likely conduits for cross-species transmission of respiratory pathogens like influenza viruses because of their close and long-term contact with their owners, audiences, domestic animals, wild animals, and birds (Figure 1, panel B) (*5*). However, the breadth and diversity of this interface presents a challenge for monitoring the emergence of infectious diseases. We have approached this challenge by conducting longitudinal studies at several sites and collecting biological samples and behavioral data representing various contexts of human–NHP contact (*4*–*7*). We used these historical and newly acquired samples, representing various countries and contexts of human–macaque contact, to determine whether NHPs are infected with influenza viruses in nature.

**Figure 1:**
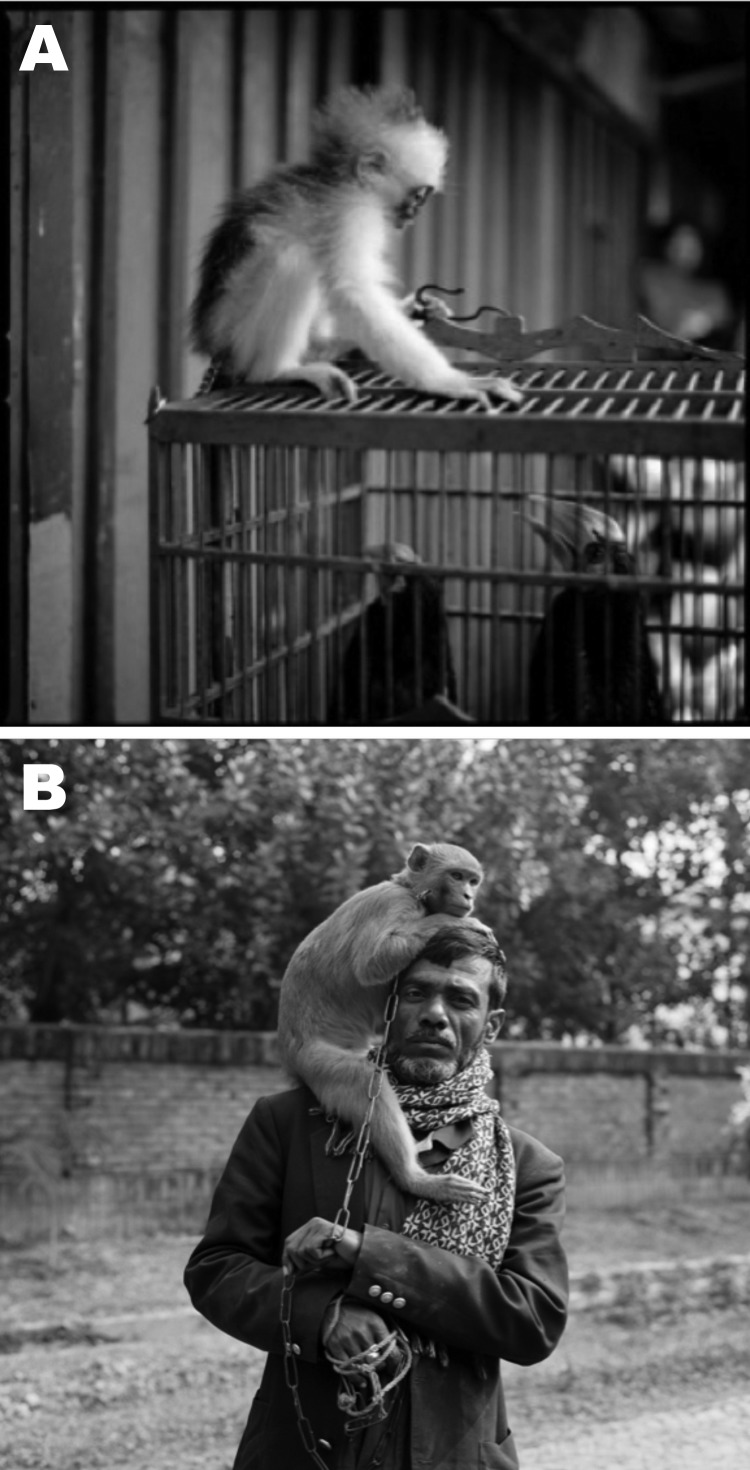
The interface between nonhuman primates, birds, and humans. A) A young, recently captured leaf monkey perched on a cage containing birds in a wet market in Java. B) A man and his performing monkey in Bangladesh. Reprinted with permission from Lynn Johnson, 2012.

## The Study

**Figure 2:**
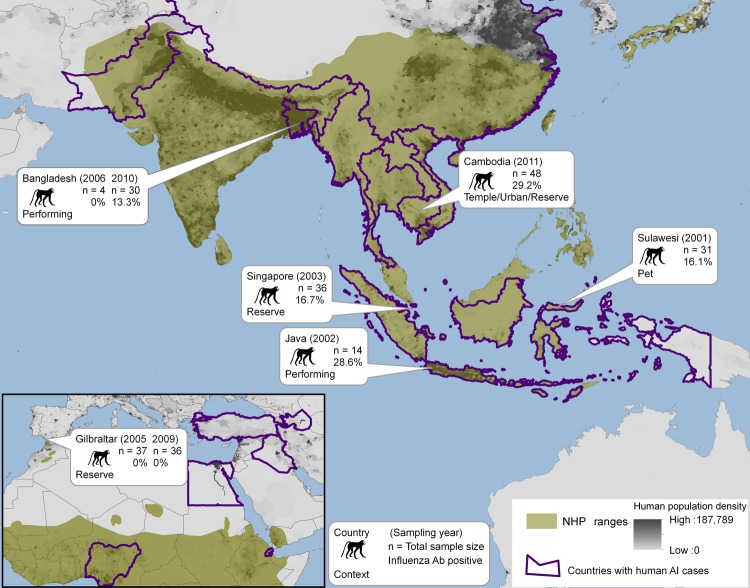
Nonhuman primate (NHP) habitat countries (in green) and approximate location of sampling sites, with sample size, year collected, context of human–macaque interaction, and seroprevalence of antibodies against influenza virus A. Countries that have reported human influenza infection of avian origin (AI) are outlined in purple.

As part of our decade-long longitudinal studies, ≈200 serum samples were collected from macaques. These included pet macaques (*Macaca nigra, M. nigrescens, M. hecki*) from Sulawesi, Indonesia; performing macaques from Java, Indonesia (*M. fascicularis*) and from Bangladesh (*M. mulatta*); *M. fascicularis* macaques from the Bukit Timah and Central Catchment Nature Reserves in Singapore, where they freely interact with wild avian fauna and visitors (occasionally entering residential areas) (*7*); *M. sylvanus* macaques from the Upper Rock Nature Reserve in Gibraltar, where international tourists frequently use food to entice the macaques to climb about their heads and shoulders (*6*); and free-ranging macaques (*M. fascicularis* and *M. nemestrina*) at temple shrines or *M. fascicularis* macaques that range throughout a wildlife rescue center and nearby villages in Cambodia (Figure 2). Serum was collected and stored as described (*8*). All samples were stored on cold packs in the field and transferred to dry ice for shipment to the United States, where they were then stored at −80°C.

For initial screening for antibodies against influenza virus, serum samples were treated with receptor-destroying enzyme as described (*9*) and tested by using a multispecies Influenza A Virus NP Antibody Inhibition Test (Virusys Corporation, Taneytown, MD, USA) according to manufacturer’s instructions. ELISA results indicated nucleocapsid protein antibodies against influenza in samples from macaques from Cambodia (29.2%), Singapore (16.7%), Sulawesi (16.1%), Bangladesh (13.3%), and Java (6.0%) (Table 1). Antibodies were detected in animals 1–10 years of age at the time of sampling. No influenza virus–specific antibodies were detected from the 73 total samples from Gibraltar, perhaps because persons with influenza virus infection infrequently travel to the Upper Rock Reserve (healthy-visitor effect) (*10*) or perhaps because monkeys from Gibraltar are less susceptible to infection. Seroprevalence of antibodies against influenza A, by site and collection year, human and NHP population densities, and prevalence of avian influenza viruses are shown in Figure 2.

**Table 1:** Anti-influenza nucleocapsid protein antibodies in nonhuman primate populations, 2001–2011*

Location, year	No.	*Macaca* species	Type(s)	No. (%) positive
Singapore, 2003	36	*fascicularis*	Reserve	6 (16.7)
Indonesia				
Java, 2002	14	*fascicularis*	Performing, pet	4 (28.6)
Sulawesi, 2001	31	*nigra*, *hecki*, *nigrescens*	Pet	5 (16.1)
Gibraltar				
2005	37	*sylvanus*	Reserve	0
2009	36	*sylvanus*	Reserve	0
Bangladesh				
2006	4	*mulatta*	Performing, pet	0
2010	30	*mulatta*	Performing, pet	4 (13.3)
Cambodia, 2011	48	*fascicularis, nemestrina*	Temple, urban, reserve	14 (29.2)

*Testing was conducted with a multispecies influenza A virus nucleocapsid protein antibody inhibition test for strain-specific antibodies.

Serum samples that were positive by ELISA were also screened by hemagglutination-inhibition assay as described (*9*). Based on the year and location of NHP sample collection, the estimated ages of the NHPs at the time of sample collection, and the presence of avian H5 and H9 influenza viruses in many of these countries during the sampling period (*11*–*13*), a panel of human vaccine strains and avian influenza virus strains was used in the hemagglutination-inhibition assay. Although not all ELISA-positive serum samples could be subtyped, antibodies against seasonal subtype H1N1 and H3N2 influenza A strains were detected from macaques in Bangladesh, Singapore, Java, and Sulawesi (Table 2). Of the performing macaques in Bangladesh, 2 had antibodies against A/chicken/Bangladesh/5473/2010, a strain of G1 clade subtype H9N2 avian influenza virus. Subtype H9N2 influenza viruses are prevalent in poultry in Bangladesh (*14*) and have been detected in humans (*12*). We did not detect antibodies against highly pathogenic avian influenza subtype H5 viruses, which might not be surprising given our relatively small sample size (Table 2). Also given the small sample size, we were unable to perform microneutralization studies, which would be useful to perform with future samples.

**Table 2:** Seroprevalence of influenza A virus subtypes in monkeys with nucleocapsid protein–positive ELISAs, by location*

Virus strain	Virus subtype (H5 clade)	Years used in vaccine	No. tested/no. positive				
			Singapore	Indonesia		Bangladesh	Cambodia
				Java	Sulawesi		
A/Beijing/262/1995	H1N1	1999–2000	0/6	2/4	1/6†	NSA	NSA
A/Sydney/5/1997	H3N2	1999–2000	2/6‡	0/4	1/6§	NSA	NSA
A/New Caledonia/20/1999	H1N1	2000–2007	0/6	0/4	1/6†	0/4	0/14
A/Panama/2007/1999	H3N2	2000–2004	2/6‡	0/4	1/6§	0/4	NSA
A/California/07/2004	H3N2	2005–2006	NSA	NSA	NSA	0/4	0/14
A/Wisconsin/67/2005	H3N2	2006–2008	NSA	NSA	NSA	0/4	0/14
A/Brisbane/59/2007	H1N1	2008–2010	NSA	NSA	NSA	1/4	0/14
A/Brisbane/10/2007	H3N2	2008–2010	NSA	NSA	NSA	0/4	0/14
A/California/04/2009	H1N1	2010–present	NSA	NSA	NSA	0/4	0/14
A/Perth/16/2009	H3N2	2010–present	NSA	NSA	NSA	0/4	0/14
A/chicken/Bangladesh/5473/2010	H9 G1	NA	NSA	NSA	NSA	2/4	NSA
A/Vietnam/1203/2004	H5 (1)	NA	NSA	NSA	NSA	NSA	0/14
A/Cambodia/R0H05050/2007	H5 (1)	NA	NSA	NSA	NSA	NSA	0/14
A/duck/Hunan/795/2002	H5 (2.1)	NA	NSA	NSA	NSA	0/4	NSA
A/BHG/Qinghai/01/2005	H5 (2.2.2)	NA	NSA	NSA	NSA	0/4	NSA
A/JWE/Hong Kong/1038/2006	H5 (2.3.4.2)	NA	NSA	NSA	NSA	0/4	NSA
A/duck/Laos/3295/2006	H5 (2.3.4.2)	NA	NSA	NSA	NSA	0/4	NSA

*Samples were only tested for relevant strains based on the collection location, year of collection, and estimated age of the monkey. Monkeys from Indonesia were not tested for influenza subtype H5N1 viruses because samples were collected in 2001 and 2002 and subtype H5N1 viruses were first reported in poultry from Indonesia in February 2004. Samples with a hemagglutination inhibition value ≥1:10 were considered positive. NSA, no samples available for testing; NA, not applicable for use in vaccine; BHG, bar-headed goose; JWE, Japanese white-eye. †Individual monkey gave positive results for both strains. ‡Individual monkeys gave positive results for both strains. §Individual monkey gave positive results for both strains.

In 2011, to determine whether any macaques were actively infected with influenza virus, we collected oral swabs from 48 monkeys in Cambodia to test for influenza virus by real-time reverse transcription PCR as described (*8*). In brief, the inside of the anesthetized and immobilized monkeys’ mouths (cheeks, tongue, and gums) were swabbed. Swabs were immediately placed into viral transport media, stored, and shipped as previously described. RNA was isolated by using an Ambion MagMAX-96 AI/ND Viral RNA Isolation Kit (Life Technologies Corporation, Grand Island, NY, USA) on a Kingfisher Flex system (Thermo Fisher Scientific, Waltham, MA, USA). Viral RNA was analyzed in a Bio-Rad CFX96 Real-Time PCR Detection System and a C1000 Thermocycler (Bio-Rad, Hercules, CA, USA) with TaqMan Fast Virus 1-Step Master Mix (Applied Biosystems, Foster City, CA, USA) and the InfA primer/probe sets as described (*15*). Of the 48 respiratory samples, 1 (2.1%) was positive for influenza virus; cycle threshold value was 38 (limit of detection is 40). Attempts to amplify longer PCR fragments of matrix, hemagglutinin, or neuraminidase genes or to isolate the virus by blind passage in embryonated chicken eggs or MDCK cells were unsuccessful.

## Conclusions

Our results indicate that NHPs that have contact with humans can be naturally infected with seasonal endemic human influenza viruses and with emerging pandemic-risk avian influenza viruses. We found serologic evidence of infection in several countries, contexts, and macaque species. Preliminary real-time reverse transcription PCR results also pointed to the presence of virus in a buccal swab from an adult macaque from Cambodia, indicating active infection at the time of sampling. On the basis of results from this study, it seems that pet, performing, and free-ranging macaques are susceptible to influenza virus infection. Given the close relationship between humans and NHPs in areas of the world where avian and human influenza viruses cocirculate, further surveillance of these populations is warranted. The ability to detect and eventually isolate strains of influenza virus currently infecting NHPs and humans at the animal–human interface is paramount to understanding how NHP–human interactions can affect the genetics, transmission, and public health risk for infection with influenza A viruses.
